# Decreased level of consciousness in acute ischemic stroke: risk factors, territories, stroke mechanisms and outcome. A single-centre cohort study

**DOI:** 10.1007/s00415-025-13548-5

**Published:** 2025-12-21

**Authors:** P. Palazzo, D. Strambo, S. Pistocchi, G. Saliou, S. Puricel, D. Lambrou, A. O. Rossetti, P. Michel

**Affiliations:** 1 Department of Clinical Neurosciences, Stroke Centre, Neurology Service, Lausanne University Hospital and University of Lausanne, Rue du Bugnon 46, 1011 Lausanne, Switzerland; 2 Stroke Unit, Riviera-Chablais Hospital, Rennaz, Switzerland; 3 Diagnostic Neuroradiological Unit, Service of Diagnostic and Interventional Radiology, Department of Medical Radiology, Lausanne University Hospital and University of Lausanne, Lausanne, Switzerland; 4 Interventional Neuroradiological Unit, Service of Diagnostic and Interventional Radiology, Department of Medical Radiology, Lausanne University Hospital and University of Lausanne, Lausanne, Switzerland; 5 Cardiology Service, Cantonal Hospital of Fribourg, Fribourg, Switzerland; 6 Department of Medicine, University of Thessaly, Larissa, Greece; 7 Epilepsy and EEG Unit, Neurology Service, Department of Clinical Neurosciences, Lausanne University Hospital and University of Lausanne, Lausanne, Switzerland

**Keywords:** Consciousness, Ischemic stroke, Wakefulness, Stroke

## Abstract

**Background:**

A decreased level of consciousness (DLOC) at onset is infrequent in patients with acute ischemic stroke (AIS). We assessed stroke localization, patient and stroke characteristics, revascularization rates, and long-term outcome in consecutive AIS patients presenting with acute DLOC.

**Methods:**

We assessed all AIS patients from 1/2003–6/2021 admitted to a single academic hospital. DLOC at stroke onset was defined as being described in the prehospital phase or being present on hospital arrival based. Using multivariable regression analyses, we compared patients with and without DLOC regarding anatomical stroke localization, baseline characteristics, frequency of revascularization, favourable functional 3-month outcome, and 12-month mortality.

**Results:**

Among 6491 consecutive AIS patients (median age = 74.8 years, IQR:20.2; 44.2% female), 778 (12%) had DLOC, representing 10.0% of patients from the primary catchment area. Strokes affecting the upper brainstem, thalamus or temporal lobes more frequently showed DLOC. DLOC was independently associated with multiple territory lesions, posterior circulation and right cerebral localization, unwitnessed stroke onset, stroke severity, initially missed stroke diagnosis (all adjusted *p*-values < 0.05). Admission temperature and systolic blood pressure showed a U-shaped association with DLOC. After multiple adjustments, favourable 3-month outcome was less frequent (adjusted OR (ORadj):0.54,95%CI:0.43–0.69) and 12-month mortality rate higher in DLOC patients (ORadj:1.35 (95%CI:1.16–1.56). The latter difference disappeared after adjusting for change of goals of care.

**Conclusions:**

The 10% of AIS patients with DLOC most often involve the upper brainstem, thalami and temporal lobes. DLOC patients have a higher likelihood of unwitnessed stroke onset and of their stroke being missed. Long-term functional outcome is clearly worse and mortality higher with DLOC even after adjustment for other prognostic factors.

**Supplementary Information:**

The online version contains supplementary material available at 10.1007/s00415-025-13548-5.

## Introduction

Decreased level of consciousness (DLOC) has been observed in 5–38% of acute stroke patients [1–4], mainly in those with thalamic lesions extending into the brainstem [5], with extensive cerebral infarcts [6] or in haemorrhagic strokes with early mass effect [7, 8]. In fact, stroke is one of the most frequent causes of consciousness disturbance in emergency rooms, together with trauma and ethanol intoxication [9].

In acute ischemic stroke (AIS), the pathophysiology of DLOC is most likely related to damage of the arousal system, which includes neurons and projections into the brainstem, thalamus, posterior hypothalamus and basal forebrain [5]. Additional potential causes are a progressive mass effect due to brain oedema or haemorrhagic transformation, encephalopathy from early infectious or metabolic complications, and epileptic seizures. Advanced age and extensive cerebral infarct were found to be risk factors for early consciousness disorder in AIS, which was associated with a higher frequency of stroke-related complications and a worse clinical outcome [6, 10]. These data, stemming from relatively small stroke cohorts from more than a decade ago, are often unadjusted for other demographic and clinical factors, and include subacute patients.

In this study, we aimed to assess in a large registry of consecutive AIS patients with DLOC the localization, stroke and patient characteristics, proportion of initially missed AIS diagnosis, and long-term outcome.

## Methods

Consecutive adult AIS patients admitted within 24 h of stroke onset or last known well time (LKW) to the stroke and/or intensive care unit of our institution from January 2003 to June 2021 were selected for this analysis. Patient data was extracted from the Acute Stroke Registry and Analysis of Lausanne (ASTRAL), a hospital-based prospective registry of consecutive AIS in a comprehensive stroke centre, as previously published [11]. This registry incorporates a large number of demographic, clinical, pathophysiological, radiological, and biological variables in a prespecified manner that we extracted and analysed for this retrospective study. Variables extracted are listed in the supplementary material.

Patients were considered as having a DLOC in the presence of a value other than zero on the National Institutes of Health Stroke Scale (NIHSS) item 1a at the first NIHSS performed within 24 h of hospital admission. This definition was considered more appropriate than the Glasgow Coma Scale (GCS) or the Full Outline of UnResponsiveness (FOUR) score because A) they may be decreased in stroke patients with focal neurological deficits such as aphasia or eye-opening apraxia, and B) NIHSS and history taking are routinely performed for all acute stroke patients at admission while GCS and FOUR scores are not. In patients arriving intubated to our centre, we reviewed all available prehospital information to ascertain that DLOC was present before intubation. In all these patients, care was taken not to confuse DLOC with eye-opening apraxia, bilateral ptosis, or locked-in state. In order not to miss patients with transient DLOC in the prehospital phase who subsequently recover normal consciousness, we also included AIS patients in the DLOC group if somnolence, stupor or coma was described by medical personnel in the prehospital phase or on hospital admission. The level of consciousness was recorded according to the status (observed by medical personnel) before potential sedation. This information is routinely collected through history taking in the ASTRAL during the acute hospitalisation phase.

Functional outcome using the mRS at 3 months, assessed either in person in the outpatient stroke clinic or by a structured telephone interview by mRS-certified personnel and mortality, assessed at 12 months using telephone interviews by mRS-certified personnel and hospital records were extracted.

### Primary and secondary outcomes

First, we calculated the proportion of patients with DLOC within all AIS admitted within 24 h of LKW to our stroke centre. We then repeated this calculation in patients arriving from the primary catchment area of our stroke centre, i.e. patients where our institution is the primary institution for patients with suspicion of stroke, as opposed to patients referred from other regions bypassing a local hospital.

Then, we defined five groups of variables as primary outcomes to assess associations in patients with and without DLOC:

- Patient and stroke characteristics, i.e. demographics, pre-stroke handicap, vascular risk factors, comorbidities, type of stroke onset, prehospital arrival time, missed/uncertain initial stroke diagnosis, basic physiological variables (admission blood pressure, temperature, glucose level), and stroke mechanism.
- Stroke localization, i.e. anatomical brain structure, right brain vs all other stroke localizations, simultaneous multiple territories, undetermined localization, and deep lacune in deep MCA structures.
- Frequency of revascularization, i.e. only Intravenous thrombolysis (IVT), endovascular treatment (EVT) (± IVT), any revascularization.
- Medium-term outcome, i.e. favourable functional outcome at 3 months defined as an mRS of 0–2 (analysis limited to patients with a pre-stroke mRS ≤ 2).
- Long-term mortality, i.e. time to death over the first 12 months after the index stroke.

Secondary outcome measures included decompressive craniectomy for ischemic mass effect in the subacute phase, change of goals of care (i.e. palliative decision during hospitalization), length of hospital stay, ordinal and dichotomized mRS at 7 days, 3 months and 12 months, and in-hospital mortality and at 3 months.

## Statistical analysis

The primary response variable was the presence of DLOC. For the entire population and each group of interest, ordinal and continuous data were summarized as medians accompanied by interquartile range (IQR), while for categorical variables counts and proportions are presented.

Thereafter, we performed a series of multivariable regression analyses (MVRAs) to identify statistically important associations of pre-selected covariates with DLOC or other response variables of interest.

For the analysis aiming to identify the relationship between DLOC and patient/stroke characteristics, we selected clinically meaningful variables in these domains including demographics, pre-stroke handicap (mRS), history of cerebrovascular events, prehospital metrics, admission NIHSS, uncertain or missed stroke diagnosis in the emergency room, arterial stroke territory, stroke mechanisms, admission temperature, systolic blood pressure and glycemia. For the association of DLOC with stroke localization, the above mentioned anatomical and vascular territory information were selected as co-variates. To assess the association of DLOC with revascularization treatments, we performed three separate MVRAs, using the significant variables from the previous analysis on patient/stroke characteristics each time and adding A) IVT yes/no; B) EVT (± IVT) yes/no, and C) any acute revascularization treatment (IVT and/or EVT vs no treatment). For the medium-term analysis of favourable outcome, variables that are known to be associated with functional stroke prognosis were selected [12, 13], such as pre-stroke mRS, admission NIHSS, age, sex, acute blood glucose, systolic blood pressure, (pc-)ASPECTS on acute imaging, history of depression or psychosis, and the use and type of acute revascularization procedure. Finally, the time to death over 12 months was displayed using a Kaplan–Meier survival curve. Then, the data was analysed using a Cox proportional hazard ratio model, with stroke death as the time-dependent explanatory variable (since inclusion in the study was at the time of the index stroke). The patients were censored at time of first recurrent event, the time of loss to follow-up, or the date of death, and all patients were censored to the right at 12 months (end of observation period). Variables known to be associated with long-term mortality after ischemic stroke were selected for adjustment [12], i.e. the same variables as in the preceding functional outcome analysis, and adding coronary artery disease, peripheral artery disease, active cancer. We finally repeated this survival analysis adding also change of goals of care.

In all MVRAs, missing values for the independent variables were imputed using multiple chain equations methodology [14]. In this way, five complete datasets were generated, and analysis of each dataset was performed separately. Stepwise selection methods were employed to identify significant associations of the selected covariates with the response, on each imputed dataset. The reported results were derived by appropriately combining the results of the five imputed analyses. The R statistical software version 4.4.0 (R Core Team 2017) was employed for all statistical analyses.

The Strengthening the Reporting of Observational Studies in Epidemiology (STROBE) approach was applied to report results [15].

## Results

A total of 6491 consecutive AIS patients were included, of whom 778/6491 (12.0%) had a DLOC in the prehospital phase and/or on hospital admission. Among patients from the primary catchment area, 404/4044 (10.0%) had DLOC; this proportion was lower than in the 374/2447 (15.3%) patients coming from other areas.

Demographic, clinical, pathophysiological, radiological, and biological variables, as well as information regarding previous cerebrovascular events and cerebrovascular risk factors are displayed in Table 1. Median age was 74.8 years (IQR 20.2), 44.2% (2871/6491) were female, and admission NIHSS was a median of 6 (IQR 11). IVT and EVT (± IVT) rates were 22.8% (1460/6399) and 17.1% (1093/6399), respectively, with his relatively high EVT rate being explained by about one third of our patients being specifically referred for revascularisation.

**Table 1:** Baseline characteristics of the overall patient population and patients with and without DLOC. Continuous and ordinal variables were expressed as medians (with interquartile range, IQR), and categorical variables as absolute counts (with percentage), unless otherwise stated. The number of missing values can be detected from the absolute counts in most instances

Variable	Overall population (*N* = 6491)	Patients with DLOC (*N* = 778)	Patients without DLOC (*N* = 5713)
Age	74.8 (20.2)	75.1 (22.5)	74.7 (19.8)
Female sex	2871/6491 (44.2%)	365/778 (46.9%)	2506/5713 (43.9%)
Private health insurance	1113/6435 (17.3%)	96/777 (12.4%)	1017/5658 (18.0%)
Vascular risk factors			
Hypertension	4763/6490 (73.4%)	547/778 (70.3%)	4216/5712 (73.8%)
Diabetes	1253/6488 (19.3%)	140/777 (18.0%)	1113/5711 (19.5%)
Dyslipidaemia	4904/6483 (75.6%)	504/775 (65.0%)	4400/5708 (77.1%)
Tobacco consumption	1504/6459 (23.3%)	153/774 (19.8%)	1351/5685 (23.8%)
Atrial fibrillation	1935/6488 (29.8%)	287/777 (36.9%)	1648/5711 (28.9%)
Coronary artery disease	1245/6482 (19.2%)	158/777 (20.3%)	1087/5705 (19.1%)
Prosthetic heart valves	255/6488 (3.9%)	37/778 (4.8%)	218/5710 (3.8%)
Low ejection fraction ≤ 35%	360/6455 (5.6%)	59/776 (7.6%)	301/5679 (5.3%)
Active cancer	335/6455 (5.2%)	35/776 (4.5%)	300/5679 (5.3%)
Migraine history	382/6443 (5.9%)	32/769 (4.2%)	350/5674 (6.2%)
Current alcohol abuse	687/6463 (10.6%)	68/773 (8.8%)	619/5690 (10.9%)
History of depression or psychosis	861/6474 (13.3)	111/778 (14.3)	751/5701 (13.2)
Body mass index	25.0 (5.0)	25.0 (6.0)	25.0 (5.0)
Previous cerebrovascular event	1818/6491 (28.0%)	183/778 (23.5%)	1635/5713 (28.6%)
Therapy at stroke onset			
Antiplatelets	2409/6485 (37.1%)	286/778 (36.8%)	2123/5707 (37.2%)
Anticoagulants	885/6459 (13.7%)	123/776 (15.9%)	762/5683 (13.4%)
Antihypertensives	3919/6482 (60.5%)	458/777 (58.9%)	3461/5705 (60.7%)
Statins	1945/6486 (30.0%)	228/778 (29.3%)	1717/5708 (30.1%)
Time metrics			
Onset-to-door time (min)	198 (510)	168 (408)	198 (522)
Baseline NIHSS on admission	6.0 (11.0)	20.0 (12.0)	5.0 (9.0)
Radiological features on admission imaging performed < 24 h			
Onset-to-CT or to-MRI time (min)	264 (570)	204 (486)	270 (576)
Acute ischemic lesion on first CT or MRI (DWI)	1990/5636 (35.3%)	222/710 (31.3%)	1768/4926 (35.9%)
Baseline (pc-)ASPECTS (acute CT or MRI)	10.0 (2.0)	9.0 (4.0)	10.0 (2.0)
Ischemic mass effect on initial imaging	7/6124 (0.1%)	3/747 (0.4%)	4/5377 (0.1%)
Symptomatic haemorrhagic transformation on initial imaging	2/6124 (0.0%)	0/747 (0.0%)	2/5377 (0.0%)
≥ 50% stenosis, occlusion or dissection in the ischemic territory on acute CTA or MRA			
Intra and/or extracranially	3462/5596 (61.9%)	587/695 (84.5%)	2875/4901 (58.7%)
Intracranially	3136/5599 (56.0%)	566/695 (81.4%)	2570/4904 (52.4%)
Extracranially	1278/5583 (22.9%)	243/695 (35.0%)	1035/4888 (21.2%)
Acute revascularization treatment			
No recanalization treatment	3846/6399 (60.1%)	366/769 (47.6%)	3480/5630 (61.8%)
Thrombolysis only	1460/6399 (22.8%)	151/769 (19.6%)	1309/5630 (23.3%)
Endovascular treatment ± thrombolysis	1093/6399 (17.1%)	252/769 (32.8%)	841/5630 (14.9%)
Stroke mechanism			
Atherosclerosis (with ≥ 50% stenosis)	975/6482 (15.0%)	125/778 (16.1%)	850/5704 (14.9%)
Cardiac	1934/6482 (29.8%)	287/778 (36.9%)	1647/5704 (28.9%)
Lacunar	658/6482 (10.2%)	14/778 (1.8%)	644/5704 (11.3%)
Dissection	231/6482 (3.6%)	43/778 (5.5%)	188/5704 (3.3%)
Undetermined	1865/6482 (28.8%)	177/778 (22.8%)	1688/5704 (29.6%)
Other determined/rare	374/6482 (5.8%)	67/778 (8.6%)	307/5704 (5.4%)
Multiple/coexisting causes	445/6482 (6.9%)	65/778 (8.4%)	380/5704 (6.7%)
Acute vital parameters and laboratory exams			
Temperature (°C)	36.3 (0.7)	36.2 (0.9)	36.4 (0.7)
Systolic blood pressure (mmHg)	151 (35)	145 (36)	151 (34)
Blood glucose (mmol/L)	6.6 (2.3)	7.2 (2.6)	6.5 (2.1)
Creatinine (μmol/L)	86 (31)	85 (33)	86 (30)
C-reactive protein (mg/dl)	4 (9)	6 (14)	3 (8)
White blood cell count (g/L)	8.2 (3.8)	9.5 (5.2)	8.1 (3.5)
Haemoglobin (g/dL)	13.8 (2.3)	13.6 (2.5)	13.9 (2.2)

^*^Based on the largest extent on both clinical and all available radiological exam grounds

*Abbreviations* CTA: Computed Tomography Angiography, DWI: Diffusion-Weighted Imaging, MRA: Magnetic Resonance Angiography, NIHSS: National Institutes of Health Stroke Scale, (pc-)ASPECTS: (posterior circulation) Alberta Stroke Program Early CT Score

**Fig. 1:**
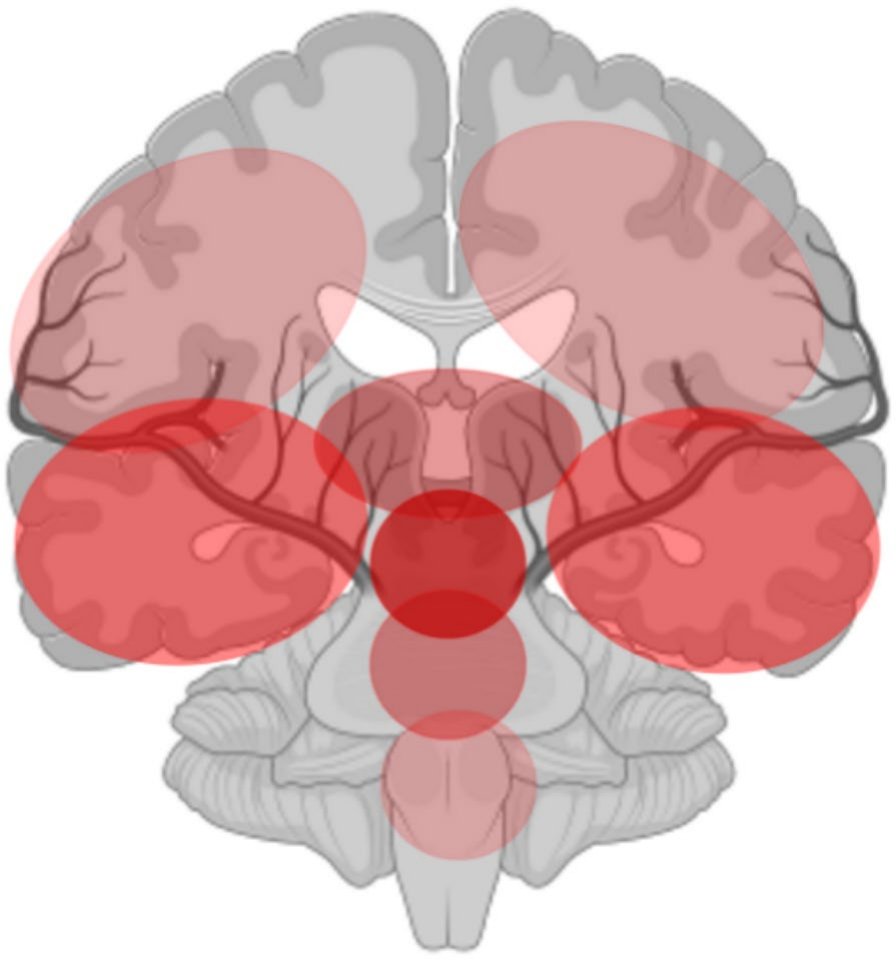
Association of DLOC with anatomical brain structures (see Table 2 for details). The darker the red colour of the anatomical structure, the stronger the association with DLOC

Several anatomical stroke localizations and vascular territories were significantly associated with a DLOC in both univariate and multivariable analyses. The absolute numbers are shown in Table 2. The MVRA for *anatomical* stroke localization showed the strongest association for diencephalic (thalamic and mesencephalic) localization, followed by the temporal lobes and the pons, as shown in Fig. 1 and Table S1. When further analysing the association of DLOC with the temporal lobe localisation, we found DLOC in 21% of patients having purely posterior circulation strokes (causing *mesio*-temporal involvement, 49/237), and in 22% of purely anterior circulation patients (causing *latero*-temporal ischemia, 432/1943).

**Table 2:** Vascular territory and anatomical structure(s) affected by the AIS. Results are given as absolute counts (with percentage), unless otherwise stated. The number of missing values can be detected from the absolute counts

Variable	Overall population (*N* = 6491)	Patients with DLOC (*N* = 778)	Patients without DLOC (*N* = 5713)
Arterial territory involved			
Anterior circulation	4404/6487 (67.9%)	499/778 (64.1%)	3905/5709 (68.4%)
Posterior circulation	1658/6487 (25.6%)	241/778 (31.0%)	1417/5709 (24.8%)
Anterior and posterior circulation	190/6487 (2.9%)	36/778 (4.6%)	154/5709 (2.7%)
Undetermined	235/6487 (3.6%)	2/778 (0.3%)	233/5709 (4.1%)
Multi-territory stroke	225/4890 (4.6%)	54/651 (8.3%)	171/4239 (4.0%)
Brain side affected			
Right only	2607/6488 (40.2%)	273/778 (35.1%)	2334/5710 (40.9%)
Anatomical structure(s) affected by the AIS *			
Frontal	3464/6035 (57.4%)	505/778 (64.9%)	2959/5257 (56.3%)
Temporal	2255/6021 (37.5%)	506/778 (65.0%)	1749/5243 (33.4%)
Parietal	2977/6020 (49.5%)	497/777 (64.0%)	2480/5243 (47.3%)
Occipital	651/6034 (10.8%)	144/778 (18.5%)	507/5256 (9.6%)
Border Zones	265/6021 (4.4%)	26/778 (3.3%)	239/5243 (4.6%)
Thalamic	614/6036 (10.2%)	168/778 (21.6%)	446/5258 (8.5%)
Mesencephalic	426/6033 (7.1%)	159/778 (20.4%)	267/5255 (5.1%)
Pontic	521/6032 (8.6%)	114/778 (14.7%)	407/5254 (7.7%)
Medulla	201/6032 (3.3%)	28/778 (3.6%)	173/5254 (3.3%)
Cerebellar	657/6033 (10.9%)	134/778 (17.2%)	523/5255 (10.0%)
Deep supratentorial lacune	630/6023 (10.5%)	39/778 (5.0%)	591/5245 (11.3%)

**Fig. 2:**
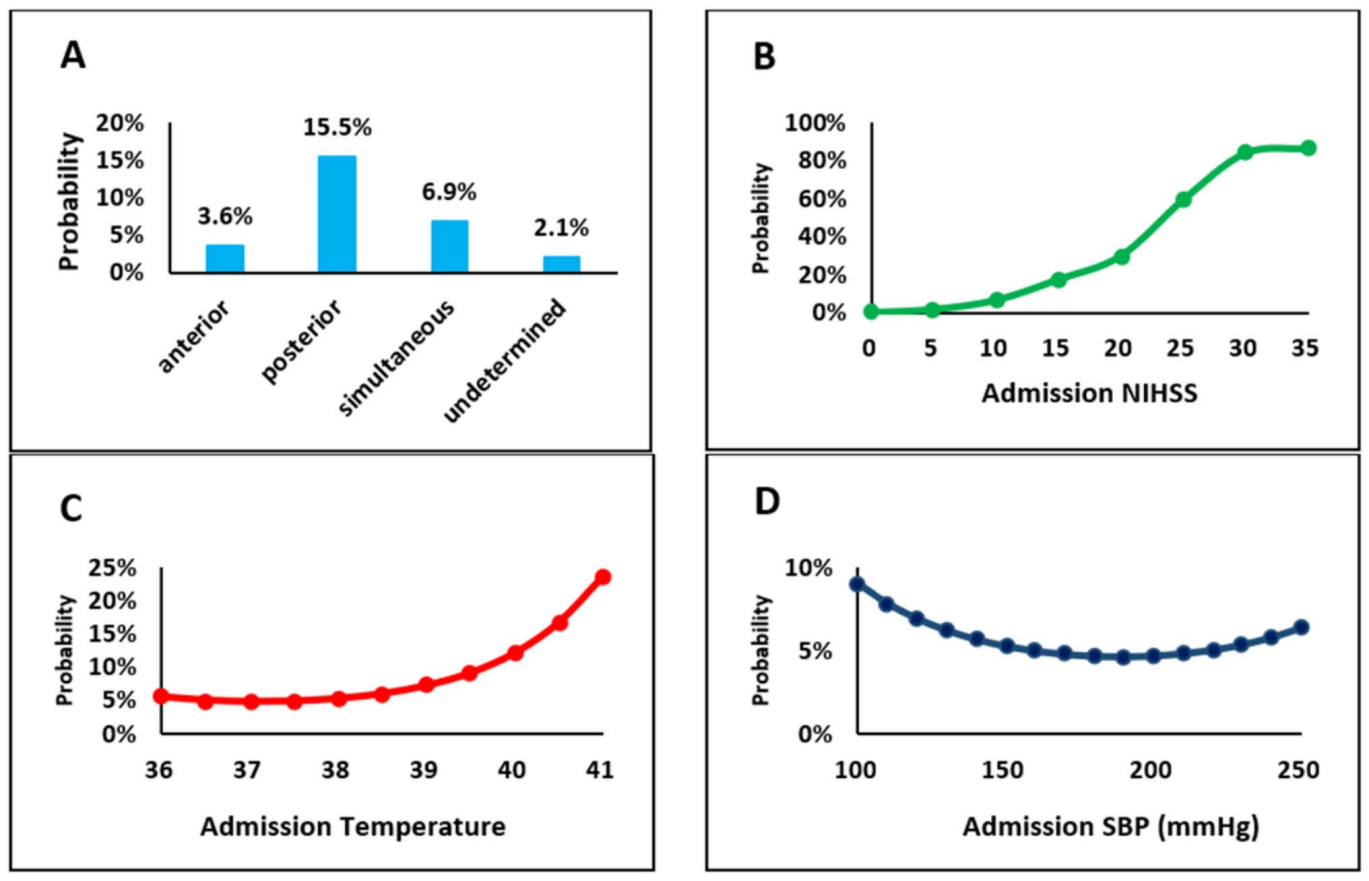
Graphical unadjusted associations of selected variables with DLOC: **A** vascular territory, **B** National Institutes of Health Stroke Scale (NIHSS), **C** temperature, and **D** systolic blood pressure (SBP)

The MVRA for *vascular territories* (adjusted for multiple demographic and clinical variables) identified the posterior circulation and multiple territory pattern as significantly more frequent in DLOC patients (Table 3 and Fig. 2A); whereas the univariable analysis showed left cerebral lesions to be more frequent in DLOC (Table 2), MVRA inversed this finding and showed an independent association between right-sided lesions and DLOC (Table 3 and Fig. 2A).

**Table 3:** Demographic, clinical variables and vascular risk factor associations with DLOC in AIS. Odds ratios and p-values are given for all the variables analysed in the unadjusted and the adjusted analyses comparing patients with and without DLOC

Variable	Univariable analysis			Multivariable analysis		
	Odds ratio	95% CI	*P* value	Adjusted log-odds ratio*	95% CI	*P* value
Onset of stroke						
Sleep (vs wake)	1.03	0.86–1.24	ns	0.07	− 0.15–0.29	ns
Unwitnessed (vs wake)	2.83	2.25–3.56	< 0.01	0.58	0.28–0.87	< 0.01
NIHSS on admission	1.19	1.18–1.21	< 0.01	Non-linear association (see Fig. 2B)		< 0.01
Vascular territory affected						
Posterior (vs anterior)	1.33	1.13–1.57	< 0.01	1.61	1.35–1.86	< 0.01
Anterior & posterior (vs anterior)	1.83	1.26–2.66	< 0.01	0.60	− 0.03–1.23	ns
Undetermined (vs anterior)	0.07	0.02–0.27	< 0.01	− 0.52	− 1.94–0.90	ns
Multiple territory pattern (vs single)	2.15	1.57–2.96	< 0.01	0.82	0.29–1.35	< 0.01
Right sided localization (vs all other possibilities)	0.78	0.67–0.91	< 0.01	0.20	0.01–0.40	0.04
Stroke not recognized (or uncertain)	2.17	1.59–2.96	< 0.01	0.94	0.50–1.38	< 0.01
Acute temperature	0.75	0.67–0.85	< 0.01	Non-linear association (see Fig. 2D)	< 0.01	
Acute systolic blood pressure	0.93	0.91–0.96	< 0.01	Non-linear association (see Fig. 2C)	< 0.01	

Variables entered in the MVRA model: age, sex, pre-stroke mRS, previous cerebrovascular events, pre-hospital metrics, admission NIHSS, uncertain or missed stroke diagnosis in the emergency room, arterial stroke territory, anatomic localization, stroke mechanisms, admission temperature, systolic blood pressure and glycemia

^*^ Adjusted results are expressed on the log-odds scale: values > 0 indicate a positive association, and below zero a negative association

*Abbreviations* CI: Confidence Interval, mRS: modified Rankin Scale, MVRA: Multivariable Regression Analysis, NIHSS: National Institutes of Health Stroke Scale

In the MVRA of *clinical variables*, we found significant association of DLOC with unwitnessed (but not in sleep) stroke onset, with missed/uncertain stroke diagnosis in the emergency room and with stroke severity as measured by the NIHSS (Fig. 2B). Among the different physiological parameters, the admission temperature and systolic blood pressure showed a U-shaped association with DLOC (Table 3, Fig. 2C and D).

We classified the circumstances of an initially missed stroke diagnosis in 42 of the 778 patients with DLOC (5.4%) in 4 categories:

- Preexisting and currently active comorbidities (such as current drug addiction, severe psychiatric pathology and epilepsy) masking the stroke diagnosis (*n* = 8)
- New comorbidities (such as sepsis, benzodiazepine overdose, massive pulmonary embolism and heart failure) (*n* = 9) masking the stroke diagnosis
- Incorrect medical reasoning (may be related to limited medical training, knowledge, or experience) (*n* = 21); in 4 of them, the stroke was also radiologically missed.
- Radiologically missed strokes (*n* = 8); in 4 of them, stroke was also missed due to other (human) factors.

Regarding the univariable *radiological analysis* (Table 1), patients with DLOC underwent imaging earlier, had somewhat lower (pc-)ASPECTS, similar and very low (< 0.5%) rates of early ischemic mass effect or haemorrhagic transformation, respectively, and higher rates of extra- and/or intracranial arterial pathology.

Rates of any type of *acute revascularization* were similar in both groups after multiple adjustments (Table 4). This was also true for rates of IVT and EVT (± IVT) separately, although there was a trend towards less IVT in patients with a DLOC (OR 0.78, CI 0.60–1.01, *p* = 0.06).

**Table 4:** Association of DLOC with acute revascularization treatments, 7-day and 3-month functional status, and 12-month mortality

Variable	Univariable analysis			Multivariable analysis		
	Odds ratio	95% CI	P value	Adjusted odds ratio	95% CI	P value
Any acute revascularization	1.78	1.53–2.07	< 0.01	0.98	0.80–1.21	ns
Thrombolysis alone	1.10	0.90–1.34	ns	0.78	0.60–1.01	ns
Endovascular treatment (± thrombolysis)	2.85	2.39–3.40	< 0.01	1.16	0.92–1.46	ns
Favourable outcome at 7 days*	0.14	0.12–0.18	< 0.01	Not analysed	Not analysed	Not analysed
Favourable outcome at 3 months*	0.17	0.14–0.20	< 0.01	0.54	0.43–0.69	< 0.01
Mortality at 12 months	4.19	3.55–4.94	< 0.01	1.35	1.16–1.56	< 0.01
Mortality at 12 months adjusted also for change in goals of care				1.12	0.97–1.31	ns

Variables entered in the MVRA model for acute revascularization treatments: age, sex, pre-stroke mRS, previous cerebrovascular events, pre-hospital metrics, admission NIHSS, uncertain or missed stroke diagnosis in the emergency room, arterial stroke territory, anatomic localization, stroke mechanisms, admission temperature, systolic blood pressure and glycemia, and acute revascularization treatments

Variables entered in the model for favourable modified Rankin score at 3 months: age, sex, pre-stroke mRS, active cancer, pre-hospital metrics, admission NIHSS, (pc)ASPECTS on acute imaging, admission systolic blood pressure and glycemia, and acute revascularization treatments

Variables entered in the model for Cox-regression of mortality over the first 12 months: age, sex, pre-stroke mRS, active cancer, coronary artery disease, atrial fibrillation, low (< 35%) ejection fraction, peripheral artery disease, pre-hospital metrics, admission NIHSS, (pc)ASPECTS on acute imaging, admission systolic blood pressure and glycemia, and acute revascularization treatments

^*^ mRS 0–2 if pre-stroke mRS ≤ 2

*Abbreviations* CI: confidence interval, mRS: modified Rankin Scale, MVRA: multivariable regression analysis, NIHSS: National Institutes of Health Stroke Scale, (pc-)ASPECTS: (posterior circulation) Alberta Stroke Program Early CT Score

**Fig. 3:**
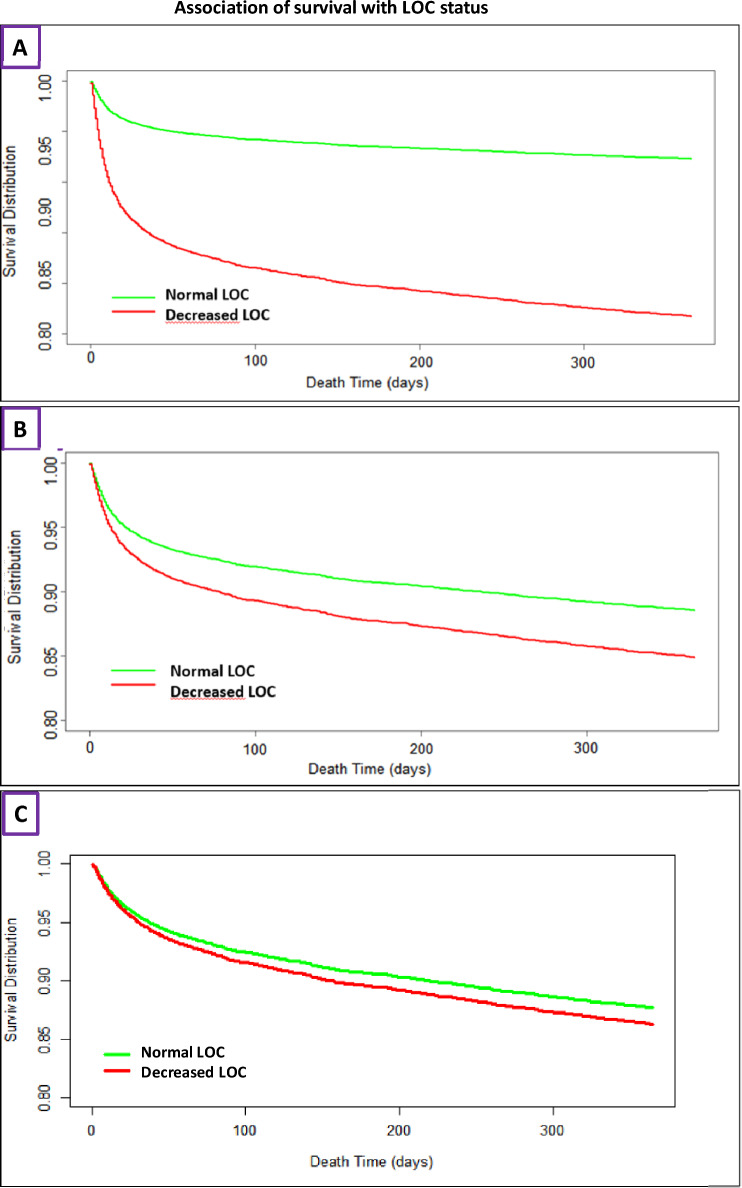
Adjusted and unadjusted survival curves over 12 months for patients with (red) and without (green) DLOC. **A** Kaplan–Meier survival curve without adjustment for other factors; **B** same, but with adjustment for multiple other prognostic factors; **C** same, but with additional adjustment for a change in goals of care LOC: level of consciousness

*Functional outcome at 3 months* in patients with premorbid mRS ≤ 2 points was favourable in 49.0% of patients with, and in 62.7% patients without DLOC. This resulted in an adjusted OR for favourable outcome of 0.54 (CI 0.43–0.69, *p* < 0.01) in presence vs absence of DLOC after adjustment as described in the methods section (Table 4). At 12 months, patients with DLOC on admission had a mortality rate of 50.4% compared to 23.5% without DLOC (*p* < 0.01), with an adjusted OR of 1.35 (95%CI 1.16–1.56, *p* < 0.01). However, when change in goals of care was added to the MVRA, DLOC became no longer significant (Table 4). Adjusted and unadjusted survival curves are shown in Fig. 3.

From the univariate analyses of the *secondary outcome variables*, we found that patients with DLOC underwent decompressive craniectomy for ischemic mass effect more often than patients without DLOC (OR 6.86, CI 4.13–11.40) (Table 4). Similarly, a change in goals of care was clearly more frequently made during the hospitalization phase in patients with DLOC (OR 6.68, CI 5.59–7.99), and they had a longer length of stay (OR 1.01, CI 1.00–1.02). DLOC patients had more unfavourable outcomes at 7 days as evaluated by dichotomized mRS (OR 0.14. CI 0.12–0.18, *p* < 0.01), as well as at 12 months (OR 0.21, CI 0.17–0.25), *p* < 0.01). They also had higher mortality rates both in-hospital (OR 20.54, CI 15.51–27.21) and at 3 months (OR 8.31, CI 6.86–10.07).

## Discussion

In our large single-centre consecutive cohort, one out of ten AIS patients had a DLOC in the prehospital phase and/or on hospital arrival when considering the primary catchment area, and 12% when adding referred patients mostly for revascularization treatments. AIS with DLOC more often had an unwitnessed stroke onset, multi-territory localization, and a U-shaped association with admission temperature and systolic blood pressure. Ischemic lesions most often affected the diencephalon and temporal lobes. Long-term functional outcome was clearly worse and mortality rate higher in these patients, independently of other prognostic factors. However, when we adjusted the mortality analysis also for the palliative care decision, DLOC was no longer significant, probably due to the self-fulfilling prophecy withdrawal bias.

Regarding lesion localization, we found a strong association with thalamic and mesencephalic structures, followed by the temporal lobes and the pons. The preferential involvement of the upper brainstem and thalamus in DLOC patients is likely related to acute damage to the arousal system. In fact, according to early models, the ascending reticular activating system (ARAS) originates from diffuse neurons in the brainstem reticular formation and includes discrete brainstem regions that are preferentially involved in maintaining a state of wakefulness [16–18]. In a study conducted on 33 stroke patients with acute occlusion of the Percheron artery, impaired arousal was correlated with lesions in the paramedian posterior thalamus, posterior hypothalamus, and midbrain tegmentum [5]. In another study on 37 patients with artery of Percheron infarction, a decreased level of consciousness was frequently observed in patients both with and without lesion extension into the midbrain [19]. Similarly, our findings are consistent with previous work linking pontine tegmentum lesions to coma in humans [16] and coma-like outcomes in animals [20]. On the contrary, our finding of the temporal lobes being particularly involved in AIS with DLOC is novel; we did not find other pathologies with temporal lobe lesions that frequently display DLOC. One hypothetical explanation for our findings is epileptic seizures, which may be subclinical [21] and tend to occur more often in strokes affecting temporal lobes [22]. Alternatively, this finding might be due to the association between the observed posterior circulation involvement and mesio-temporal ischemia, the latter territory also being supplied by the posterior circulation. However, our findings of DLOC being present in similar frequencies in patients with purely posterior vs anterior circulation stroke (21 and 22%), do not support this hypothesis.

We also found right cerebral lesions to be more frequent in DLOC patients than in controls. Although we cannot exclude that this association is at least in part related to misinterpretation of eye-opening apraxia as DLOC, we paid careful attention in clinical practice to differentiate between the two clinical signs. Reasons why right-sided cerebral lesions are more frequent in patients with DLOC are not evident.

Finally, the finding that multiple territory strokes are associated with DLOC is not surprising as multiple lesions may affect the projections of the ARAS into both hemispheres.

We also found that stroke severity was also associated with DLOC. This finding is in part tautological, given that the NIHSS includes assessment of the level of consciousness; we decided to keep the NIHSS in the MVRA to correct for other clinical associations. This finding may of course also be explained by the association of clinical stroke severity with infarct size, as shown by Li et al. [23] Secondly, unwitnessed (but not in sleep) onset correlated with DLOC; this may be interpreted as people living alone and having severe strokes with DLOC, therefore being unable to indicate the precise onset of stroke and activate the medical alert system.

We also found that DLOC at stroke onset is associated with an increased risk of not recognizing stroke, which is often due to “stroke chameleons”, i.e., DLOC being misdiagnosed as toxic-metabolic encephalopathy or traumatic brain injury leading to missed or delayed revascularization treatment as shown in previous studies [24–27].

Among the different physiological parameters, we showed a U-shaped association of DLOC with low and high temperatures and systolic blood pressure. This cannot be explained by extreme physiological values worsening the ischemic stroke alone as our analysis was adjusted for stroke severity. These physiological parameters might indicate decreased capacity of the non-ischemic brain to compensate for the acute stroke-related neurological deficit.

We had expected increased age or pre-existing disability to be associated with more DLOC because such patients might have less brain reserve to compensate for initial neurological deficits. One potential explanation for the absence of such an association was our statistical adjusting for stroke severity. Another negative finding was the lack of association between DLOC and specific stroke mechanisms which was somewhat unexpected given that cardioembolic strokes tend to be more severe, and lacunar strokes, less severe [11]. Again, the integration of the baseline NIHSS in the multivariable analysis may have corrected for such findings.

Acute neuroradiological abnormalities were more pronounced in DLOC patients, as expected. On the other hand, there was a very low (< 0.5%) rate of early ischemic mass effect or haemorrhagic transformation, most likely given the short median time to first neuroimaging of less than 6 h; such findings did therefore not therefore explain DLOC in our population.

The fact that we did not find a difference in acute revascularization rates between DLOC and control patients after multiple adjustments is reassuring because the presence or absence DLOC should not by itself preclude revascularization treatment. The numerically (but not statistically significant) lower IVT rate could partially be explained by the higher rate of initially unrecognized stroke diagnosis in the DLOC group or by delays in acute management because of the need to stabilize or intubate such patients.

The observation of worse functional and survival outcomes at 3 months in patients with DLOC despite adjustment for multiple other prognostic factors is consistent with previous research [10, 27]. This might be the result of DLOC indicating more severe stroke independently of the NIHSS, of more neurological and internal medicine complications in DLOC patients, and of initial diagnostic errors or delays.

Our finding that patients with DLOC underwent decompressive craniectomy for ischemic mass effect more often is likely explained by the fact that DLOC is associated with the large initial stroke volume [28, 29]. Our observation that palliative care decisions were made more frequently in patients with DLOC can be explained by the repeatedly shown unfavourable prognostic value [10, 30].

Strengths of the study are its reliance on data collected in one of the most detailed registries of consecutive AIS patient data stemming from routine clinical practice.

Our study has several limitations. The definition of DLOC always contains a subjective element of the observer; although standardized and validated scales such as the GCS or FOUR scales exist, their limitations are well known in particular in acute stroke patients with language problems [31]. Also, we relied in a subset of patients arriving with normal LOC on clinical observation in the prehospital phase; we decided to do so in order to capture also patients with rapidly regressive DLOC, and limited false observations by considering only prehospital reports by medical personnel (typically paramedical). We also did not collect data on the degree of DLOC (somnolence, stupor, coma) in the prehospital phase (if transient) or on arrival (if persistent). We did not measure certain variables that could induce DLOC in AIS such as acute encephalopathy related to metabolic problems or infections; we did however include acute blood sugar level and assess acute temperature; both were not related to DLOC. Also, we did not routinely record (EEG) and search for epileptic seizures or status epilepticus in the acute phase of these AIS patients. Although clinically rare in our AIS population [32] we found “subclinical” epileptiform activity in the subacute phase of ischemic stroke in 16% of continuously monitored acute stroke patients [21]. Finally, this is a retrospective, observational quality control study conducted in a single tertiary stroke centre, with a predominance of central European, elderly white patients. Our findings, therefore, might not be generalizable to other populations or settings.

In conclusion, in a large consecutive monocentric cohort, patients with DLOC in the acute phase of AIS more often had upper brainstem, thalamic and temporal lobe stroke localization, multi-territory strokes, an unwitnessed stroke onset, stroke being uncertain or missed initially, and worse long-term functional and mortality outcomes.

## Supplementary Information

Below is the link to the electronic supplementary material.

Supplementary file1 (DOCX 39 KB)

## Declarations

### Data Availability

We will make supplementary files and key datasets openly available in appropriate digital data repositories that conform to the Fair Data principles and maintained by a non-profit organisation to demonstrate reproducibility. Unstructured data will be shared via the H2020 data repository Zenodo-FBM/CHUV community.
