# Supplementary material for: Decreased level of consciousness in acute ischemic stroke: risk factors, territories, stroke mechanisms and outcome. A single-centre cohort study
Source: J Neurol. 2025 Dec 21;273(1):35. doi: 10.1007/s00415-025-13548-5 (PMC12719347; doi:10.1007/s00415-025-13548-5)
Supplement: Supplementary file 1 — Supplementary file1 (DOCX 39 KB) [file 415_2025_13548_MOESM1_ESM.docx]

**Supplementary methods: Variables extracted from the Acute Stroke Registry and Analysis of Lausanne (ASTRAL) and analysed in the current study**

Variables extracted included age, biological sex and pre-stroke disability estimated by the modified Rankin scale (mRs). We also registered information on previous cerebrovascular events and cerebrovascular risk factors such as hypertension, hyperlipidaemia, diabetes mellitus, tobacco consumption, atrial fibrillation, prosthetic heart valves, low cardiac ejection fraction ≤35%, active cancer (i.e. known active or actively treated cancer, or cancers discovered during the index stroke admission or within the following 12 months), migraine history, current alcohol abuse, and body mass index.

Strokes were reported as the vascular territory (anterior, posterior, both, multiple, undetermined), the anatomical brain structures affected, and the side of the brain, using the largest extent on both clinical and all available radiological exam grounds.

Process-oriented data were collected in the prehospital phase (known stroke onset vs wake-up stroke vs unwitnessed onset, pre-hospital time between onset (or last known well) and hospital arrival). The collected National Institutes of Health Stroke Scale (NIHSS) at admission are evaluated by either NIHSS-certified or supervised by NIHSS-certified personnel.

We collected vital signs (skin temperature, blood pressure) and acute blood glucose, measured on admission. Admission brain imaging (CT and/or MRI) was reviewed for the presence or absence of ischemic changes, the Alberta Stroke Program Early CT Score (ASPECTS)^1^ or pc-ASPECTS^2^ in posterior circulation stroke, ischemic mass effect, and early haemorrhagic transformation. CT-angiography or MR-angiography on admission were reviewed for significant arterial pathology intra- and extracranially, i.e. for occlusion or ≥ 50% stenosis within the ischemic territory.

Stroke mechanism was extracted, classified according to the TOAST trial,^3^ with three further categories added: “cervical artery dissection”, “rare determined mechanisms”, and “multiple/coexisting causes”.

Acute revascularization treatment data was collected, performed according to the in-hospital guidelines that are constantly adapted according the most recent national^4 5^ and international^6-8^ guidelines and trial results.

**Table S1:** Anatomical localisation of DLOC in AIS. Odds ratios and p-values are given for the unadjusted and the adjusted analyses comparing patients with and without DLOC for 12 variables with localisation information.

| Localisation | Univariable regression analysis | | | Multivariable regression analysis | | |
| --- | --- | --- | --- | --- | --- | --- |
|  | Odds ratio | 95% CI | P value | Odds ratio | 95% CI | P value |
| Frontal | 1.44 | 1.23-1.68 | <0.01 | 2.03 | 1.46-2.82 | <0.01 |
| Temporal | 3.72 | 3.17-4.35 | <0.01 | 6.67 | 5.09-8.76 | <0.01 |
| Parietal | 1.98 | 1.69-2.31 | <0.01 | 1.77 | 1.25-2.50 | <0.01 |
| Occipital | 2.13 | 1.74-2.61 | <0.01 | - | - | ns |
| Thalamic | 2.97 | 2.44-3.62 | <0.01 | 7.51 | 5.23-10.78 | <0.01 |
| Mesencephalic | 4.80 | 3.88-5.94 | <0.01 | 7.49 | 5.34-10.51 | <0.01 |
| Pontine | 2.04 | 1.64-2.56 | <0.01 | 4.34 | 3.10-6.08 | <0.01 |
| Medulla oblongata | 1.10 | 0.73-1.65 | ns | 3.01 | 1.75-5.18 | <0.01 |
| Cerebellar | 1.88 | 1.53-2.32 | <0.01 | 2.25 | 1.67-3.02 | <0.01 |
| Deep supratentorial | 0.42 | 0.30-0.58 | <0.01 | 1.59 | 1.08-2.34 | 0.02 |
| Undetermined localisation | 0.07 | 0.02 - 0.30 | <0.01 | - | - | ns |
| Right-sided stroke vs all other | 0.78 | 0.67 - 0.91 | <0.01 | - | - | ns |
| Interaction term temporal-mesencephalic | - | - | - | 0.24 | 0.12-0.48 | <0.01 |
| Interaction term temporal-thalamic | - | - | - | 0.26 | 0.15-0.46 | <0.01 |

Variables entered in the MVRA model: each anatomical brain structure, deep supratentorial small size stroke, undetermined localisation, side of stroke.

Abbreviations: CI: confidence interval, MVRA: multivariable regression analysis.

**Table S2:** Cox regression analyses for 12-month mortality, without (1) and with (2) the variable “change in goals of care”

| Variable | Multivariable analysis 1 | | | Multivariable analysis 2 | | |
| --- | --- | --- | --- | --- | --- | --- |
|  | Odds ratio | 95% CI | P value | Adjusted odds ratio | 95% CI | P value |
| Age | 1.04 | 1.03-1.04 | <0.01 | 1.02 | 1.01-1.02 | <0.01 |
| Endovascular treatment (±thrombolysis) | 0.72 | 0.62-0.83 | <0.01 | ns | | |
| Thrombolysis alone | 0.86 | 0.75-1.00 | <0.05 | ns | | |
| Cancer | 2.93 | 2.47-3.47 | <0.01 | 2.31 | 1.94-2.74 | <0.01 |
| Pre-stroke mRS | 1.32 | 1.26-1.39 | <0.01 | 1.14 | 1.08-1.19 | <0.01 |
| NIHSS on admission | 1.09 | 1.08-1.10 | <0.01 | 1.05 | 1.04-1.06 | <0.01 |
| ASPECTS on acute CT scan | 0.91 | 0.89-0.93 | <0.01 | ns | | |
| SBP in the acute phase | 0.98 | 0.95-1.00 | <0.05 | 0.96 | 0.94-0.98 | <0.01 |
| Blood glucose | 1.04 | 1.03-1.06 | <0.01 | 1.05 | 1.03-1.06 | <0.01 |
| DLOC | 1.35 | 1.16-1.56 | <0.01 | 1.12 | 0.97-1.31 | ns |
| Atrial fibrillation | 1.17 | 1.03-1.32 | <0.05 | 1.28 | 1.13-1.44 | <0.01 |
| Coronary artery disease | 1.22 | 1.07-1.39 | <0.05 | ns | | |
| Peripheral artery disease | 1.57 | 1.31-1.87 | <0.01 | 1.44 | 1.21-1.71 | <0.01 |
| Palliative care decisions | Not included | | | 17.34 | 14.88-20.13 | <0.01 |

Variables entered in the model for these Cox-regression analyses are described in the methods section.

Abbreviations: CI: confidence interval, ns: not significant, mRS: modified Rankin Scale, NIHSS: National Institutes of Health Stroke Scale, ASPECTS: Alberta Stroke Program Early CT Score, CT: computed tomography, SBP: systolic blood pressure, DLOC: decreased level of consciousness.

1. Barber PA, Demchuk AM, Zhang J, Buchan AM. Validity and reliability of a quantitative computed tomography score in predicting outcome of hyperacute stroke before thrombolytic therapy. ASPECTS Study Group. Alberta Stroke Programme Early CT Score. *Lancet* 2000;355(9216):1670-4. doi: 10.1016/s0140-6736(00)02237-6

2. Puetz V, Sylaja PN, Coutts SB, et al. Extent of hypoattenuation on CT angiography source images predicts functional outcome in patients with basilar artery occlusion. *Stroke* 2008;39(9):2485-90. doi: 10.1161/strokeaha.107.511162 [published Online First: 20080710]

3. Adams HP, Jr., Bendixen BH, Kappelle LJ, et al. Classification of subtype of acute ischemic stroke. Definitions for use in a multicenter clinical trial. TOAST. Trial of Org 10172 in Acute Stroke Treatment. *Stroke* 1993;24(1):35-41. doi: 10.1161/01.str.24.1.35

4. Michel P AM, Hungerbühler H, Nedeltchev K, Georgiadis D, Müller F, Bönig L, Müller M, Städler C, Cereda C, Ghika J, Baum-gartner R, Sztajzel R, Weder B, Mattle HP, Lyrer P. . Thrombolyse de l’attaque cérébrale ischémique. *Forum Med Suisse* 2009;9(49):892-96.

5. Michel P, Diepers M, Mordasini P, et al. Acute Revascularization in Ischemic Stroke: Updated Swiss Guidelines. *Clinical and Translational Neuroscience* 2021;5(1):9.

6. Turc G, Bhogal P, Fischer U, et al. European Stroke Organisation (ESO) - European Society for Minimally Invasive Neurological Therapy (ESMINT) Guidelines on Mechanical Thrombectomy in Acute Ischaemic StrokeEndorsed by Stroke Alliance for Europe (SAFE). *Eur Stroke J* 2019;4(1):6-12. doi: 10.1177/2396987319832140 [published Online First: 20190226]

7. Turc G, Tsivgoulis G, Audebert HJ, et al. European Stroke Organisation - European Society for Minimally Invasive Neurological Therapy expedited recommendation on indication for intravenous thrombolysis before mechanical thrombectomy in patients with acute ischaemic stroke and anterior circulation large vessel occlusion. *Eur Stroke J* 2022;7(1):I-xxvi. doi: 10.1177/23969873221076968 [published Online First: 20220217]

8. Berge E, Whiteley W, Audebert H, et al. European Stroke Organisation (ESO) guidelines on intravenous thrombolysis for acute ischaemic stroke. *Eur Stroke J* 2021;6(1):I-lxii. doi: 10.1177/2396987321989865 [published Online First: 20210219]
